# Integrated Sensor-Optics Communication System Using Bidirectional Fiber and FSO Channels and Hybrid Deep Learning Techniques

**DOI:** 10.3390/s23208434

**Published:** 2023-10-13

**Authors:** Amare Mulatie Dehnaw, Yibeltal Chanie Manie, Li-Yuan Du, Cheng-Kai Yao, Jun-Wei Jiang, Bing-Xian Liu, Peng-Chun Peng

**Affiliations:** Department of Electro-Optical Engineering, National Taipei University of Technology, Taipei 10608, Taiwan; mulatieamare7@gmail.com (A.M.D.); yibeshmamaru@gmail.com (Y.C.M.); yasd0366@gmail.com (L.-Y.D.); t109658093@ntut.org.tw (C.-K.Y.); t111658093@ntut.org.tw (J.-W.J.); t111658015@ntut.org.tw (B.-X.L.)

**Keywords:** fiber Bragg grating, strain, free space optics (FSO), remote sensing, optical sensor system, intensity wavelength division multiplexing, central wavelength detection, sensing signal detection, deep learning, hybrid stacked gated recurrent units and long short-term memory (SGRU-LSTM)

## Abstract

This paper introduces a new bidirectional integration approach that combines fiber sensor/free space optics (FSO) communication using an intensity and wavelength division multiplexer (IWDM) techniques-based long-distance fiber Bragg grating (FBG) sensor strain-sensing system. By implementing coarse wavelength division multiplexing (CWDM), the system achieves the simultaneous transmission of optical communication and fiber optical sensor (FOS) sensing signals, resulting in a highly capable, flexible, and cost-effective solution. The proposed FSO transmission technique addresses complex fiber cable installation concerns with topographical limitations. This bidirectional structure ensures the reliability and stability of the long-distance FBG sensor system, supported by extensive research and experimentation. A hybrid stacked gated recurrent units and long short-term memory (SGRU-LSTM) model is proposed to enhance strain measurement accuracy by predicting and measuring the central wavelength of overlapped strain-sensing FBG sensor signals. The results demonstrate the superiority of the proposed model in peak wavelength detection accuracy. The primary benefit of integrating communication and sensing is the significant reduction in construction costs by eliminating the requirement for two individual fiber optic systems, as the integration allows for a single system to fulfill both functions, resulting in more efficient and cost-effective implementation. Overall, this paper contributes to advancing long-distance FBG sensor systems by integrating fiber sensor/FSO communication and deep learning techniques, improving transmission distance, multiplexing capacity, measurement accuracy, system survivability, and cost-effectiveness.

## 1. Introduction

Over the recent several decades, fiber optic sensors have undergone rapid development and emerged as a promising technology with widespread use for measuring various physical and chemical parameters [1,2,3]. These sensors utilize optical fibers as the detecting element, allowing them to detect changes in temperature, strain, pressure, and other quantities. The optical fibers are modified to enable the measurement of these parameters and are connected to a light source for detection purposes. Fiber optic sensors are commonly used in industrial settings for their ability to function in tight spaces and provide precise measurements. Optical fiber sensors have become increasingly important in various industries due to their reliability, sensitivity, and versatility in measuring various physical and chemical parameters.

In remote sensing applications, one type of sensor that has been widely employed is the fiber Bragg grating (FBG) sensor. This highly versatile sensor is extensively utilized in various applications to monitor and track an array of physical parameters. These parameters include slope, strain, pressure, acceleration, temperature, displacement, and load. These measurements are crucial for gathering valuable data in a remote sensing setting [2,4]. Additionally, the FBG sensor has emerged as a highly prospective technology within the IoT realm. This sensor offers the unique advantage of multipoint sensing, allowing for the precise measurement of a wide range of parameters. It excels in monitoring infrastructure and performing structural health monitoring (SHM) in diverse applications [2,3,4,5,6]. The FBG sensor effectively collects data from objects and accurately measures changes in the environment, ensuring high signal quality and providing valuable features [4,6]. When comparing FBG sensors to conventional electrical and mechanical sensors, it becomes evident that FBG sensors offer a multitude of advantages [4,7]. These include their inherent resistance to electromagnetic interference (EMI), high resolution, precise detection and measurement of physical parameters, lightweight design, high sensitivity, high multiplexing capacity, the ability to integrate multiple sensors into a single fiber, and low installation complexity and cost. These exceptional features of FBG sensors position them as the preferred choice for a wide range of applications in industries such as aerospace, oil and gas, civil engineering, and telecommunications, where precise and reliable sensing capabilities are imperative [4,7,8].

In these above-mentioned industrial setting applications, various parameters need to be measured using FBG sensors in remote areas, including vibration, strain, current, pressure, temperature, deformation, acceleration, voltage, and humidity [4,7,8]. These physical parameters are obtained by stretching or compressing the fiber, which modifies the refractive index and the periodic distance between the FBG sensor’s gratings. Additionally, the high multiplexing capacity of FBG sensors enables the simultaneous measurement of multiple parameters [7,8,9,10,11,12]. However, the design of a high-capacity, cost-effective, and reliable network relies heavily on the choice of multiplexer techniques and the transmission channel. Among the available multiplexer techniques for FBG sensor systems, the intensity and wavelength division multiplexer (IWDM) method is widely recognized as one of the most superior approaches. It allows for the multiplexing of FBGs into multiple arrays with a simple structure and a wide range of operational wavelengths [4,7,8]. However, the IWDM approach can create unmeasurable gaps and cross-talk between neighbor FBGs when the bandwidth of one FBG slightly exceeds that of its neighbor. To address this issue, several machine learning algorithms have been proposed [4,7,10,13,14,15]. However, it is crucial to consider that these algorithms do have inherent limitations specifically related to their training speed and learning capacity, which should be carefully evaluated and accounted for to ensure their effective implementation and successful outcomes.

On the other hand, in the integration of optical communication and sensor networks, challenges arise concerning the flexibility and reliability of the network when installing fiber cables in geographically challenging areas, like mountains, rivers, rocks, and other obstacles, to transmit communication and sensing signals in long-distance transmission systems. Fiber cables are commonly used as the standard transmission medium for long-distance communication due to their ability to provide high data rates and minimal signal loss in both rural and urban areas [4,7,16,17]. However, there are certain drawbacks associated with the use of fiber cables. These include high maintenance costs, the presence of non-linear effects, and challenges in overcoming geographical obstacles. Despite their advantages, it is important to consider these drawbacks when implementing fiber cables for communication purposes. To address these challenges, researchers have proposed the use of free space optics (FSO) transmission channels even in high atmospheric turbulence [17]. The emergence of FSO transmission channels has provided a solution to these geographical constraints, such as mountains, rivers, and other obstacles, enabling the transmission of optical and sensing signals [17,18]. FSO technology is a method that utilizes light to transmit data through the atmosphere or free space. It offers several advantages, including secure spectrum licenses, high transmission speeds, low power consumption, cost-effectiveness, and ease of deployment [19,20]. However, FSO is typically limited to short-distance communication due to its vulnerability to atmospheric turbulence. To combine the benefits of both fiber and FSO transmission channels, there have been proposals for integrating these technologies. This integration offers a promising solution for creating reliable, flexible, cost-effective networks supporting optical access and sensor network technologies.

This paper presents a novel bidirectional communication system that integrates fiber and FSO technology with an IWDM base FBG sensor network. The system utilizes a coarse wavelength division multiplexing (CWDM) multiplexer/demultiplexer (CWDM Mux/Demux) to enable the simultaneous transmission of optical communication signals and sensing signals. The utilization of CWDM technologies within an integrated fiber and FSO transmission channel serves as a cornerstone in the design of a cost-effective and flexible network, facilitating an increase in the coverage of transmission distances [21]. Simultaneously, the implementation of a bidirectional structure of fiber and FSO transmission channels is vital in reducing signal loss, particularly in the context of long-distance FBG sensor systems [22]. This proposed bidirectional structure integration of a combined fiber sensor and optical communication system also addresses the issue of signal loss that occurs after 25 km of single-mode fiber (SMF) due to Rayleigh backscattering (RB). The integration aims to enhance reliability and extend the transmission distance of the fiber/FSO communication system with the sensor network, reaching remote optical network units (ONUs) and remote FBG sensing systems. CWDM Mux/Demux technology possesses an impressive capacity when it comes to effectively combining or separating signals that are traditionally considered incompatible, as reported by researchers references [23,24]. This remarkable capability of the CWDM Mux/Demux makes it an efficient and reliable technique for seamlessly integrating communication and an FBG-based sensor system. As a result of this integration, the overall system achieves notable advantages in terms of its cost-effectiveness, high data capacity, and exceptional adaptability, as further evidenced by the aforementioned research references [23]. Thus, employing the CWDM Mux/Demux technology as a vital component in this integrated system not only ensures optimal signal compatibility and functionality but also contributes to the overall efficiency, capacity, and adaptability of the entire system architecture.

To address the signal detection issues of the FBG sensor mentioned earlier, a hybrid deep learning approach is proposed. This approach integrates two powerful deep learning techniques, namely the hybrid stacked gated recurrent units and long short-term memory (SGRU-LSTM). By integrating these two models, the hybrid approach aims to improve the accuracy and effectiveness of signal detection. The SGRU model offers the capability to capture complex temporal dependencies, while the LSTM model excels at capturing long-term dependencies in the data. This combination of models creates a synergistic effect, enabling the better handling of signal detection challenges in the FBG sensor. This innovative hybrid deep learning approach shows promising potential for enhancing the performance and capabilities of the FBG sensor in various practical applications. These combined algorithms are selected for their simplicity, strong generalization ability, high tolerance to input noise, and faster training process [25]. These qualities make it well-suited for addressing the signal detection issues in the FBG sensor. The simplicity of the algorithm allows for easy implementation and understanding, reducing the likelihood of errors. Its strong generalization ability enables it to effectively handle various types of data and adapt to different scenarios, making it a versatile solution. The algorithm’s high tolerance to input noise ensures reliable performance even in the presence of noisy data. Additionally, the faster training process enables quicker development and deployment of the model. Overall, these factors contribute to the algorithm’s suitability for addressing the signal detection issues in the FBG sensor. The experimental results validate the successful integration of optical fiber/FSO communication and the FBG sensor system. This integration enables the efficient multiplexing of sensors, simultaneous transmission of communication and sensing signals, and the capability to overcome geographical limitations.

## 2. Conceptual Structure of the Bidirectional Integration of the Fiber/FSO Sensor System

**Figure 1:**
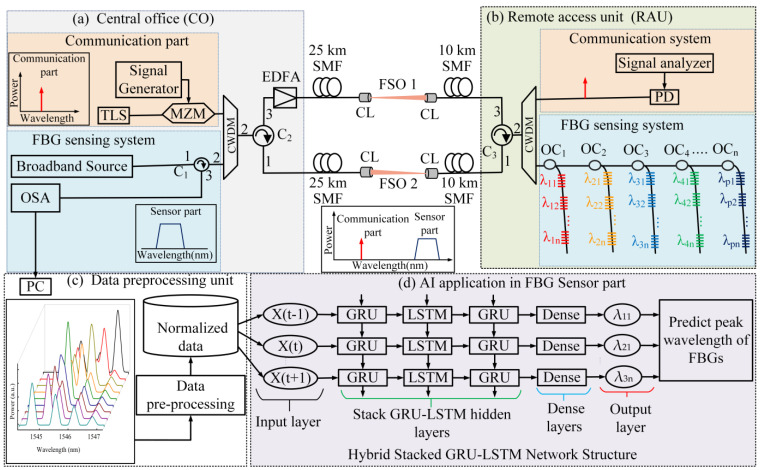
Conceptual and experimental setup structure of bidirectional integration of fiber/FSO sensor system: (**a**) central office (CO), (**b**) remote access unit (RAU), (**c**) data preprocessing, and (**d**) hybrid deep learning model.

Figure 1 depicts the schematic diagram of the proposed bidirectional communication integrated IWDM-based sensor network, which aims to enhance communication reliability within the sensor network. As shown in the figure, the bidirectional structure consists of the central office (CO), a deep learning application, and a remote access unit (RAU) that encompasses both the communication component and the remote sensing system. The CO includes a tunable laser source (TLS), broadband light source (BLS), Mach–Zehnder modulator (MZM), optical amplifier (OA), four FBG sensors, optical signal analyzer (OSA), optical circulators (C), photodetector (PD) (for communication applications), and, for the FBG sensing system, a personal computer (PC) (i.e., light source and receivers), CWDM Mux/Demux, and an artificial intelligence (AI) application. The CO is responsible for generating light sources for both the communication and sensor components. FBG sensor components, using IWDM-based techniques, can be implemented into real-world objects such as dams, bridges, gas and oil industries, and other sectors. This integration enables the effective monitoring and measurement of various parameters, facilitating accurate and reliable data collection for improved operations and maintenance. Within this conceptual framework, the utilization of the FSO link is crucial. It serves the purpose of simplifying the installation process and overcoming geographical obstacles, such as wide rivers and large mountains, where laying fiber cables is impractical. The FSO transmission channel acts as an alternative solution, circumventing the limitations of fiber optics while requiring less labor, time, and cost without the need for specific licenses. Furthermore, the measured reflected spectra or sensing signals obtained through FBG sensor components are subsequently analyzed using the implementation of an AI application. This analysis is carried out to assess the health conditions of the objects, thus enhancing the overall monitoring capabilities of the system. Within the proposed conceptual structure, the signal undergoes transmission using CWDM techniques, serving both the communication and sensor parts. Additionally, the sensor part connects to a network capable of expanding to accommodate pxn FBG sensors. This design enables the simultaneous distribution of both the communication and sensor parts of the signal to multiple remote ONU and FBG sensors. As a result, it facilitates the monitoring and measurement of diverse parameters across the ONU and sensor network. The integration of such a system allows for enhanced efficiency and effectiveness in data collection and analysis.

### Experimental Setup of the Bidirectional Integration of the Fiber/FSO Sensor System

Based on the conceptual structure in Figure 1 is the experimental configuration for integrating bidirectional optical fiber/FSO communication and a long-distance FBG sensor system using CWDM. The experimental setup consists of four main components, namely the CO, RAU, data preprocessing unit, and a hybrid stack GRU-LSTM network structure. The CO is composed of various devices including a TLS, BLS, MZM, OA, OSA, C, and CWDM. The CO is responsible for managing the light source and monitoring sensing information. The RAU component includes devices such as C, CWDM, FBG sensors, OC, and PD. The third component is the data preprocessing unit, which is responsible for data preparation and normalization for further analysis purposes. The experimental setup also incorporates a deep learning segment, which is responsible for signal detection. This segment involves several devices, such as TLS, BLS, MZM, OA, four FBG sensors, OSA, four OC, three C, 25 km and 10 km SMF, two collimator lenses, and two CWDMs. The overall setup enables the efficient integration and simultaneous transmission of optical communication signals and sensor data.

The TLS-generated light is utilized to transmit signals in the optical communication segment, while the BLS is employed to illuminate the sensor array. In this research paper, a 2 m free space optical (FSO) transmission distance is employed between two collimator lenses (CL) due to the limitation of laboratory space. These collimator lenses consist of collimators and doublet lenses. The main purpose of this paper is to demonstrate the proof of the concept through a simplified experiment. In the proposed communication and sensing system, a bidirectional MF access network is used, with a signal transmission distance of 25 km before utilizing FSO and 10 km after FSO. Within the FSO setup, the two collimator lenses are separated by a 2 m transmission distance in a free atmospheric turbulence environment, which is necessary due to the available laboratory space. Furthermore, to overcome the impact of atmospheric turbulence in free space optical (FSO) communication systems, a variety of mechanisms have been proposed. These mechanisms include approaches such as wavelength division multiplexing (WDM), buffering, retransmission, reconfiguration, and re-routing. In the context of this study, the experiment is conducted in an environment where the atmospheric turbulence effect is absent. This favorable condition allows for the transmission of data using the FSO transmission channel over distances of up to 1 km, as indicated by previous research [26]. Both the sensor and communication signals are transmitted through the FSO link, starting from the first CL and ending at the second CL. The signal transmitted by the second CL in the FSO1 link continues to propagate through another 10 km SMF toward C3. Upon reaching C3, the signal enters another device that utilizes CWDM technology. The signal output from C3 is then split into the communication and sensor signals using CWDM technology. The sensor signals are directed to the four multiplexed fiber FBG sensors in the sensing unit. On the other hand, the communication signal continues to propagate toward a photodetector (PD), where it is analyzed for parameters such as error vector magnitude (EVM) and constellation diagram.

Simultaneously, the sensing signal is distributed into an optical coupler, such as OC_1_, OC_2_, OC_3_, and OC_4_, based on IWDM techniques, and the optical power propagates toward the λ_11_, λ_21_, λ_31_, and λ_41_ sensors. Furthermore, the reflected signal is combined with the second CWDM and enters C3, where it takes a lower-side alternate route back to the CO. The signal is transmitted over a 10 km SMF, followed by the second FSO transmission channel, and another 25 km SMF before entering C2. From there, it propagates into the first CWDM through the remaining output port of C2. Subsequently, the first CWDM splits the signal, directing the reflected signal to C1, which allows it to propagate into the optical spectrum analyzer (OSA) within the FBG sensor system. The integration of the FBG sensor system and OSA data is achieved by capturing the reflected spectra of the four FBGs using Python code on a PC. Finally, the captured reflected spectra data can be transferred to the PC for further analysis and training of the proposed stack GRU-LSTM model. In the experimental setup, the integrated OF/FSO communication system incorporates four FBG sensors based on the IWDM technique. These sensors operate by exploiting the wavelength-dependent characteristics of the periodic refractive index within the core of the optical fiber. When there is a strain-induced change in the fiber’s refractive index, it leads to a shift in the sensor’s wavelength. These sensors occupy distinct wavelength ranges, allowing for the precise and simultaneous measurement of various physical parameters, such as temperature, and strain-sensing measurements. In the proposed scheme to address the issues of signal power loss and ensure a reliable output signal in long-distance FBG sensor systems, a bidirectional fiber-based structure is implemented. This approach enables alternative signal transmission in both directions, optimizing data acquisition reliability. Additionally, the incorporation of the FSO transmission technique is crucial for overcoming topographical constraints. FSO technology utilizes optical signals to transmit data through the atmosphere, offering an alternative solution in areas where wired or fiber optic networks are impractical. By using FSO, FBG sensor systems can overcome geographical obstacles, ensuring wireless communication links and data transfer over long distances without a physical infrastructure. Moreover, FSO enhances security and provides immunity from interference, making it suitable for applications requiring data privacy and integrity. The integration of a bidirectional fiber-based structure and FSO transmission technique ensures reliable and uninterrupted data transfer for accurate monitoring and analysis in various industries and applications. This integration minimizes signal loss and Rayleigh scattering. Moreover, in the proposed scenario, there is no issue with the mentioned problem. This is because the wavelength used in the communication part is different from the wavelength used in the sensing part. Additionally, the adjacent channel isolation is more than 30 dB, ensuring effective separation and minimal interference between neighboring channels. During the experiment, it was found that the wavelengths collected in the communication part were different from those in the sensing part. Therefore, this difference did not affect the normalization process or the prediction performance of the proposed GRU-LSTM method on the sensor system. To ensure the accurate and reliable estimation of sensing signals, the experimental process involves several crucial steps. Firstly, the reflected spectra data obtained from the sensors are recorded and stored for further analysis. Subsequently, these spectra data undergo preprocessing to enhance their quality and prepare them for the subsequent stages. This preprocessing step may include techniques such as reorganizing, normalization, and feature extraction. Once the data are appropriately preprocessed, hybrid deep learning models are employed to analyze and estimate the sensing signals based on the input spectra data. The data preprocessing and integration of the hybrid stacked GRU-LSTM deep learning technique are illustrated in Figure 1c,d, highlighting the significance of this approach in the experimental setup. In the experiments, the spectra of the sensors, namely λ_11_, λ_21_, λ_31_, and λ_41_, are sampled at 2001 points within a specific wavelength range of 1544 nm to 1548 nm.

## 3. The Proposed Methodology

### Data Collection and Data Preprocessing

The data collection process is conducted in a real experiment using the proposed structure. The experiment described in this research is conducted using the experimental setup illustrated in Figure 1. During the experiment, the reflected fiber grating spectra are observed and analyzed using an OSA. This allows for the visualization and monitoring of the spectral characteristics of the fiber grating. To generate the training and testing sensor spectral datasets, specific parameters are recorded within the experimental setup. These parameters include the peak power of each FBG, the wavelength shift range, and the number of sample points. During the data collection phase, consecutive strain steps are systematically applied to the λ_11_ sensor. This process enables the collection of strain-sensing data for a total of four FBGs. It is important to note that while the strain is applied to the λ_11_ sensor, causing its central wavelength to shift within a specified range, the central wavelengths of the other FBGs remain fixed. The FBG sensor data collected during the experiment are sampled at a total of 2001 sample points, covering a wavelength range of 1544 nm to 1548 nm. This particular wavelength range is carefully chosen to encompass the relevant spectral information required for accurate strain sensing.

**Figure 2:**
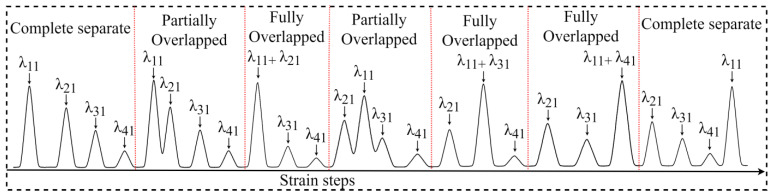
Spectral data from non-overlap to overlap and then to non-overlap as strain increases.

Figure 2 visually presents the sampled spectra obtained from the experiment. It demonstrates various situations that occur during the strain process, ranging from the original spectrum to cases of gradual partial overlap, complete overlap, and complete separation. These observed scenarios provide valuable insights into the behavior and response of the FBG sensors under different strain conditions and enhance the understanding of the strain-sensing process. By conducting this experiment and accurately recording the associated data, a deeper understanding of the behavior and performance of FBG sensors can be gained. This knowledge contributes to the advancement of strain-sensing technology and facilitates further research in this field.

During the data collection process, these strain spectral data are collected for both the training and testing proposed in the subsequent prediction model. Accordingly, 263 and 25 strain steps spectral data are collected for training and testing sets, respectively, for the proposed prediction deep learning model. Once the spectral data are collected, preprocessing techniques are applied to obtain a well-prepared dataset and to train subsequent prediction models. Data preprocessing is a crucial step in the data analysis pipeline that helps to ensure the accuracy and reliability of the results obtained from data analysis. The primary goal of data preprocessing is to improve the quality of the data, remove inconsistencies, and ensure that the data is in a format that can be easily used by data analysts and machine learning algorithms. The spectral datasets are arranged based on input features spectra and target values of each record. Data normalization is an important preprocessing step in deep learning. There are several reasons why normalization is important for deep learning models. First, normalization can help the model converge faster during training by avoiding large weight updates. Second, normalization can help prevent the saturation of activation functions, which can occur when the input values are too large. Finally, normalization can help make the model more robust to changes in the input distribution, which can improve the generalization performance of the model. Normalization refers to scaling the input features to a standard range. Next, feature scaling of relevant attributes is required to perform, which enhances the efficiency of the fitting to the prediction model. Consecutively, normalization is also one of the important data preparation techniques that can effectively reduce the loss function and speed up the gradient descent to find the optimal solution. To normalize the data, the min-max normalization method is computed in Equation (1) as follows [27]:

(1) $$y_{norm}=\frac{y-y_{min}}{y_{max}-y_{min}},$$

where y_norm_ is the normalized dataset feature, y is the original dataset feature, y_min_ is the minimum value in the y feature, and y_max_ is the maximum value in the y feature. This MinMaxScaler is the most common normalization technique. Using the MinMaxScaler standardization technique, all of the feature values are changed between 0 and 1. Subsequently, once the data preprocessing and normalization phases have been completed, the entire 263 strain spectral data are then provided to the proposed method to train a hybrid GRU and LSTM network. This approach combines the strengths and capabilities of both GRU and LSTM models, enabling enhanced performance and flexibility in handling complex features of data. The detailed operating principle and functioning of this hybrid model will be elaborated in the following section.

## 4. The Proposed Stacked GRU-LSTM Model Training

The primary objective of the proposed method is to improve the measurement accuracy in the FBG sensor system. This is achieved by predicting and determining the central wavelength of each sensor through the detection of overlap spectra and the evaluation of detection errors. This method aims to address the limitations of traditional signal processing techniques by using advanced deep learning models, such as GRU and LSTM networks. These models are capable of capturing complex relationships in sequential data-based regression, learning the features of the sequential inputs, making them suitable for accurately determining the central wavelength of FBG sensors [28,29]. By enhancing the detection accuracy of wavelength measurements, the proposed method contributes to the overall performance and reliability of the FBG sensor system, enabling its application in various domains, including structural health monitoring and industrial process control.

In this work, a hybrid stacked GRU-LSTM model is proposed to predict the central wavelength sensor from the overlap spectra, measure the detection error, and improve the measurement accuracy sensor system. Stacked GRU-LSTM is a type of neural network architecture that combines both GRU and LSTM layers in a stacked manner. GRU and LSTM are both types of recurrent neural networks (RNNs) that are commonly used for processing sequential data, such as text, speech, or time series data.

The GRU is a simpler variant of the LSTM, which was introduced to reduce the number of parameters and computational complexity of the LSTM. Both GRU and LSTM layers use gates to selectively forget or remember information from the previous time step, which helps the model handle long-term dependencies in sequential data. In a stacked GRU-LSTM architecture, multiple layers of GRU and LSTM are stacked on top of each other, with the output of each layer fed as input to the next layer. This allows the model to learn more complex and abstract representations of the input data, as each layer can capture different levels of abstraction. Multi-layer neural networks are a common powerful approach to improving a model’s performance in machine learning [30,31]. By stacking multiple layers of neurons, these models can learn increasingly complex representations of data, leading to better accuracy and performance. One popular type of multi-layer model is the deep neural network, which typically consists of multiple layers of densely connected neurons. Each layer in the network takes in the output of the previous layer and uses it to compute a new set of outputs. By combining these layers, deep neural networks can learn intricate and highly non-linear relationships between input and output data. Moreover, adding more layers to a neural network can help the model learn more complex representations of the data and can lead to better accuracy and predictive results.

**Figure 3:**
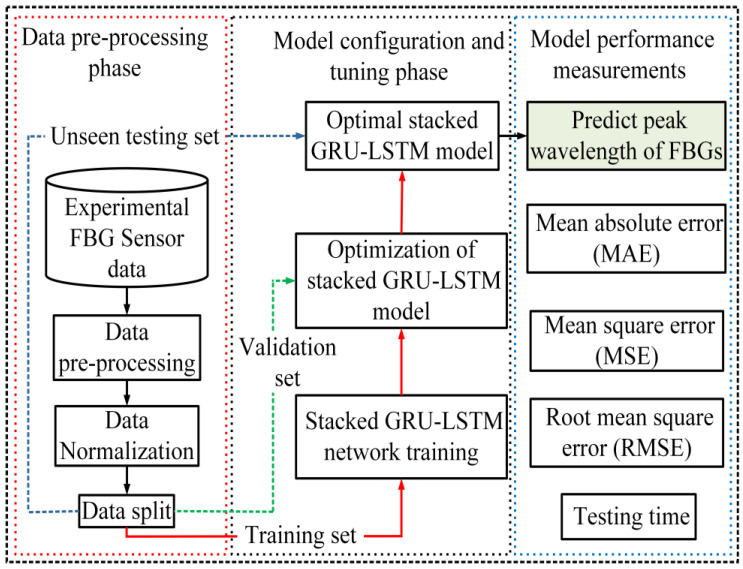
Overall system structure and training procedure of the proposed hybrid stacked GRU-LSTM methods to detect the strain sensor signals.

As illustrated in Figure 3, once real experiment-based collected spectral training datasets are preprocessed and normalized, the data are split into a training set and an unseen testing set. In deep learning applications, the validation set and unseen test set both play crucial roles in evaluating the performance of a trained model, but they operate in different stages and with distinct datasets. The validation dataset is a key component utilized during model training. It is typically a subset of the labeled dataset that is partitioned specifically for validation purposes. During the training process, the model is trained on a separate training dataset, and the validation dataset is used to assess the model’s performance after each epoch or iteration. The validation data allow for the evaluation of model performance on data that it has not seen during training. This evaluation helps in monitoring the model’s progress, fine-tuning hyperparameters, and making adjustments to improve its performance.

On the other hand, unseen datasets are entirely independent and not used during both the training and validation stages. It is carefully preserved and serves as a final evaluation of the trained model’s effectiveness in real-world scenarios. The unseen test data should be representative of the data the model might encounter after deployment. By evaluating the model on this unseen test data, researchers and practitioners can obtain an unbiased evaluation of the model’s generalization and capability to perform on new, previously unseen instances. Generally, the goal of utilizing both the validation and unseen dataset is to ensure that the trained model performs well not only on the data it was trained and validated on but also on new, unseen instances. By using the validation dataset for iterative improvements and the unseen test dataset for final evaluation, practitioners aim to build reliable and robust models that can effectively handle real-world challenges. Before the proposed stacked GRU-LSTM network training started, the training set spectral data were split into an 80:20 ratio of training and validation sets. Accordingly, the training process of the stacked GRU-LSTM network is initiated by initializing the GRU layer to receive the training samples of all four FBG spectra sequentially as input features. During the training of the proposed stacked GRU-LSTM network, several crucial parameters were carefully adjusted to optimize the performance of the model. These involved the most suitable activation function, optimizer algorithm, batch size, number of iterations (or epochs), and hidden units. By iteratively tuning these parameters, the model aimed to achieve the highest possible accuracy and effectively capture the underlying patterns within the FBG spectra.

**Figure 4:**
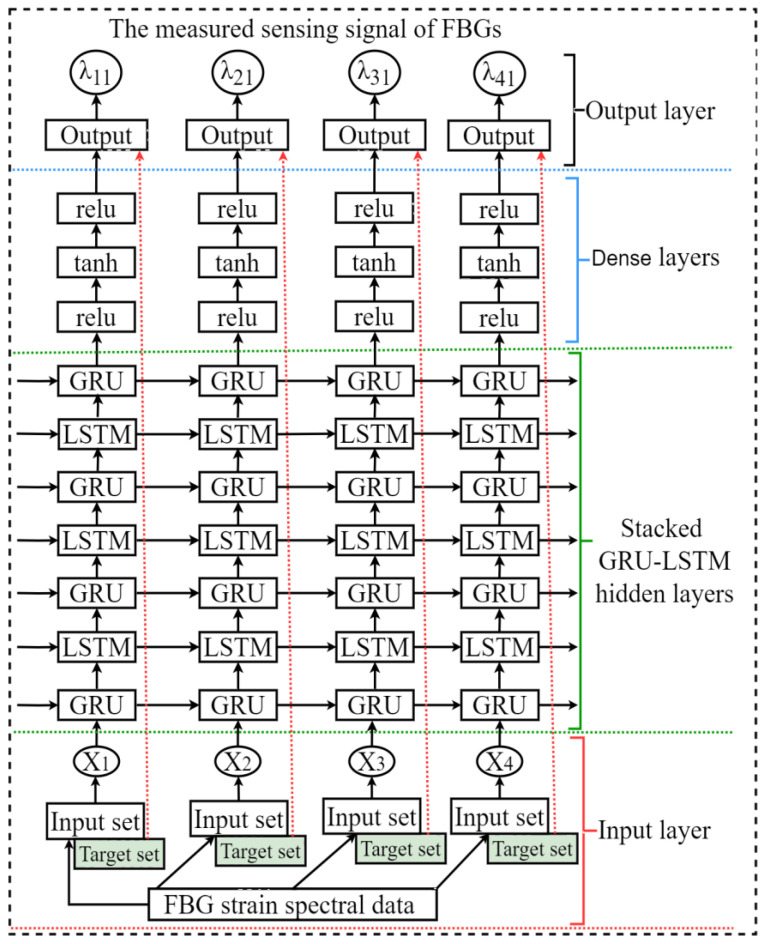
Proposed hybrid Stacked GRU-LSTM model structure.

Figure 4 demonstrates the proposed hybrid stacked GRU-LSTM architecture, which incorporates four GRU and three LSTM stacked layers. The basic architectural structure of our designed Stacked GRU-LSTM network operates as follows: It utilizes the GRU layers to handle the sequential data, capturing and retaining relevant information from preceding time steps. The output of the GRU layers is then passed on to the LSTM layers, which further process the information by incorporating memory cells and gates to manage the flow of information. This combined architecture allows for the enhanced modeling and understanding of time-dependent data, facilitating more accurate predictions and useful insights.

As shown in Figure 4, the basic architectural structure of the proposed stacked GRU-LSTM network involves several key steps. Firstly, the training data comprising reflection spectra of FBGs at different strain steps are collected and formatted into a training dataset. The figure provides a visual representation of the inputs and target values used in the proposed hybrid stacked GRU-LSTM model. To prepare the data for the model, a series of preprocessing and data structuring steps are applied. These steps ensure that the inputs and target values adhere to the required formats for deep learning. In this particular case, the target values correspond to the peak wavelength of the utilized four FBGs. These peak wavelength values are crucial in the context of the model, as they serve as the desired outputs or targets for prediction. The inputs, on the other hand, consist of the reflected spectra of the same FBGs. By analyzing these reflected spectra, the hybrid stacked GRU-LSTM model can learn to capture the underlying patterns and relationships that enable the accurate prediction of the peak wavelength values. Overall, the preprocessing and data structuring steps facilitate the proper alignment of the inputs and target values, enabling the hybrid stacked GRU-LSTM model to effectively learn and make accurate predictions in the given scenario.

Accordingly, the various parameters, including epochs, hidden layers, batch sizes, optimizers, and activation functions, are adjusted to find the optimal values. To enhance the prediction accuracy of the peak wavelength for FBGs and evaluate estimation error, the proposed model undergoes multiple parameter adjustments until it achieves optimal values. During training, various activation functions, such as Tanh, ReLU, softmax, and sigmoid, are employed to aid the convergence of the hybrid stacked GRU-LSTM model’s output. These activation functions play a crucial role in introducing non-linear features to the model. Through extensive training and evaluation using different activation functions, it is determined that both the ReLU and Tanh activations consistently yield lower RMSE values compared to other functions. Consequently, the optimal model incorporates both ReLU and Tanh activations to effectively capture non-linear characteristics and improve performance in peak wavelength prediction for the FBGs. Once the model is developed, it is tested on unseen test datasets to evaluate its performance and generalization capabilities. The prediction outputs generated by the network are compared against the actual values using a loss function, with metrics such as mean squared error (MSE), mean absolute error (MAE), and root means squared error (RMSE) used to evaluate the prediction performance.

Furthermore, the effectiveness of the stacked GRU-LSTM model is compared to other algorithms to validate its superiority. By following this architectural structure and conducting comprehensive evaluations, valuable insights into the prediction capabilities of the stacked GRU-LSTM network for FBG reflection spectra analysis are gained. This analysis allows for the assessment of the network’s performance and highlights its strengths and advantages in this domain.

**Figure 5:**
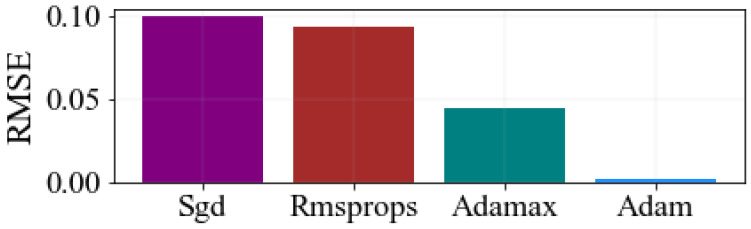
Training of hybrid stacked GRU-LSTM algorithm using real experiment spectral training sets to select optimal optimizer.

In the training phase of the stacked GRU-LSTM network, the selection of the most suitable optimizer holds great significance in assessing the performance of the proposed model. To evaluate and determine the model’s effectiveness, various evaluation metrics, such as MSE, MAE, and RMSE, are employed in the regression models. These evaluation metrics play a vital role in comparing and contrasting the performance of different variations of the stacked GRU-LSTM model, allowing for the identification of the model that proves superior performance. These performance evaluation metrics, MSE, MAE, and RMSE, are computed in Equations (2)–(4) [32], respectively, as follows:

(2) $$MSE=\frac{1}{r}\sum_{i=1}^{r}(a_{i}-{\bar{p})}^{2},$$

(3) $$MAE=\frac{1}{r}\sum_{i=1}^{r}\left|\left(a_{i}-\bar{p}\right)\right|,$$

(4) $$RMSE=\sqrt{\frac{\sum_{i=1}^{r}(a_{i}-{\bar{p})}^{2}}{r}},$$

where the number of test data values is specified by r, a_i_ means the actual central wavelength of the FBG sensor measured by OSA, and $\bar{p}$ is the estimated center wavelength output of the FBG by the proposed system. The smaller the values of the RMSE, MSE, and MAE, the lower the model’s wavelength interrogation error. Therefore, to identify the best optimizers, the proposed stacked GRU-STM network is trained repetitively. To compare the proposed model performance of different optimizers, the loss values are compared as shown in Figure 5.

As demonstrated in the figure, these optimizers achieved different RMSE values. As seen in the figure, the RMSE value of Adam is lower. Through extensive analysis and comparison, it has been determined that the Adam optimizer consistently produces the lowest RMSE values in comparison to the other optimizers. Therefore, the decision has been made to select the Adam optimizer as the optimal choice for training the proposed hybrid stacked GRU-LSTM model. The selection of the Adam optimizer is better able to facilitate the rapid convergence of both the training and validation loss, resulting in reduced RMSE values. In direct comparison with alternative optimizers, the Adam optimizer has proven to be highly effective in achieving superior performance and minimizing RMSE values [8]. To enhance training efficiency and introduce non-linearity, the network is compiled using the Adam optimization algorithm, along with the ReLU and Tanh activation functions. In the proposed stacked GRU-LSTM model, GRU and LSTM hidden units are combined to accurately measure the strain-sensing signal of the FBGs. During the training process, various parameters such as the number of epochs, hidden units, hidden layers, optimizer, and activation function are tuned to optimize the model’s performance. As a result, the proposed algorithm produces different training times, as well as varying values of RMSE, MAE, and MSE.

**Figure 6:**
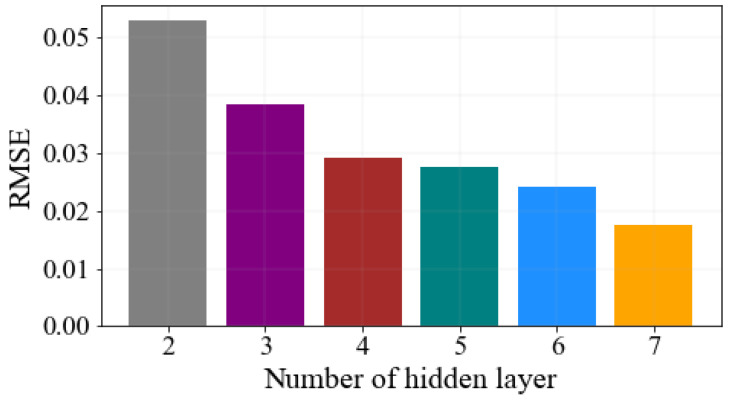
Stacked GRU-LSTM model performance number of layers with RMSE.

To identify the optimal value, several numbers of training are implemented on the proposed model until the optimal results are achieved. As a result, the optimal values are achieved in 200 batch size, 1800 epochs size, seven hidden layers, and 824 hidden units. Moreover, the proposed stacked GRU-LSTM network is compiled using the Adam optimizer, ReLU, and Tanh activation functions. In addition to this, the performance of the proposed model is evaluated by training the network using a different number of layers. When the number of layers increases in a neural network, the prediction accuracy and ability to learn more complex features can be better. Figure 6, demonstrated the performance of the stacked GRU-LSTM model in terms of RMSE and a different number of layers. As shown in the figure, when the number of stacked GRU-LSTM network layers increases, the model significantly increases the learning capability and reduces the RMSE values. This indicates the performance of the proposed model is boosted when the number of layers increases. Thus, the proposed stacked GRU-LSTM model has achieved better performance at seven layers compared to the two layers, three layers, four layers, five layers, and six layers stacked GRU-LSTM model performance.

## 5. Results and Discussion

In this chapter, a new bidirectional integration of fiber sensor/FSO communication with an IWDM-based long-distance FBG sensor system is proposed. This integration allows for the simultaneous transmission of optical communication and sensing signals using CWDM technology. The integration of communication and sensing systems offers a distinct advantage by significantly reducing construction costs. In the absence of an integrated communication and sensing system, two separate fiber optic systems would be required. However, through the integration of these functionalities, only one fiber optic system is needed. This integration empowers the system to concurrently support communication and sensing, resulting in notable cost savings. Consolidating communication and sensing into a single system brings multiple cost-effective benefits. It eliminates the need for additional infrastructure and equipment, reducing material and installation costs. The simplified system architecture makes managing and maintaining easier. An integrated system allows for optimized resource allocation. With separate systems, redundant resources are needed. Integration enables more efficient resource utilization, leading to cost reductions. In general, integrating communication and sensing is a cost-effective solution. It reduces construction costs, optimizes resource utilization, and streamlines workflows, maximizing the benefits of simultaneous capabilities. The results of this research are discussed by categorizing them into two distinct cases, each highlighting different aspects of the proposed system.

In the first case, which pertains to an IWDM-based long-distance FBG sensor system, a hybrid stacked GRU-LSTM algorithm was proposed to predict the central wavelength of the FBG sensor system. By implementing this hybrid deep learning algorithm, the peak wavelength of the FBG sensor was effectively deep learning model predicted, and the detection errors were estimated. To evaluate the performance of the proposed hybrid stacked GRU-LSTM model, several performance metrics were utilized, including loss or MSE, accuracy, MAE, RMSE, and testing time.

**Figure 7:**
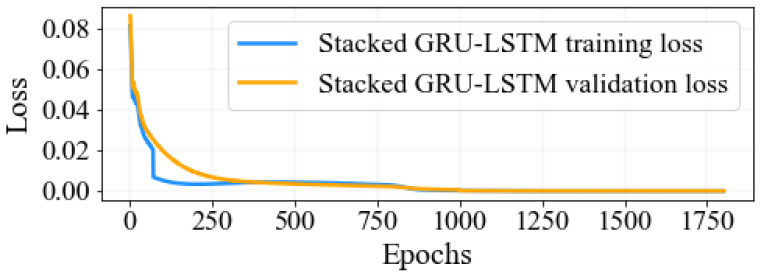
Hybrid stack GRU-LSTM model training and validation loss using real experiment spectral training sets.

According to the results illustrated in Figure 7, the training and validation losses of the proposed stacked GRU-LSTM method are illustrated as a consistent decrease with an increase in the number of epochs. The figure demonstrates that the training and validation losses started to converge after reaching 750 epochs; however, their stability is not achieved until 1000 epochs.

It is the optimal result that even though the training and validation losses had completely converged after 1300 epochs, the optimal values for these losses were obtained at 1800 epochs. This suggests the importance of prolonging the training process to achieve the best possible results. Additionally, the performance of the proposed stacked GRU-LSTM network was evaluated by training it using real experiment spectral training datasets. The evaluation aimed to determine the minimum model detection error values. Accordingly, the model achieved impressive results, with the minimum MSE value recorded at 0.00046015 and the minimum RMSE value recorded at 0.02145102. These outcomes highlight the effectiveness of the proposed approach in accurately predicting spectral data and minimizing the error between predicted and actual values. The use of real experiment spectral training datasets further enhances the reliability and applicability of the proposed stacked GRU-LSTM network in practical scenarios.

**Figure 8:**
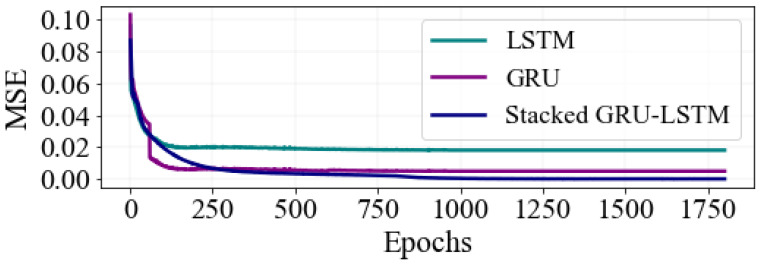
Performance comparison of the training MSE between stacked GRU-LSTM, GRU, and LSTM models.

To further validate the proposed model’s ability to detect wavelengths, a comparison was made between the peak wavelength detection performance of the hybrid stacked GRU-LSTM approach and other deep learning methods, like GRU and LSTM. This comparison was carried out using an identical training dataset size and within the same experimental working environment. As depicted in Figure 8, when the models are individually trained using real experiment-based spectral datasets, the MSE values consistently decrease with the increasing of several epoch sizes. Interestingly, the results demonstrate that the stacked GRU-LSTM model outperformed the other models, demonstrating its superior accuracy in wavelength detection. These findings provide compelling evidence of the effectiveness of the stacked GRU-LSTM model in accurately detecting wavelengths. This further validates the effectiveness and superiority of the proposed approach in wavelength detection tasks. The outcomes of the proposed hybrid model are presented to demonstrate its effectiveness in predicting the central wavelength of FBG sensors and estimating the detection error.

The performance evaluation metrics provide valuable insights into the accuracy and reliability of the hybrid stacked GRU-LSTM model. Moreover, the proposed hybrid stacked deep learning is validated even though the power variations occurred within the λ_11_, λ_21_, λ_31_, and λ_41_ sensor wavelengths. Therefore, the power variations had no significant impact on the proposed AI system. The testing time was also considered to assess the efficiency of the proposed model in real-world applications. Overall, this chapter provides a comprehensive analysis of the bidirectional integration of fiber sensor/FSO communication with the IWDM-based long-distance FBG sensor sensing system, highlighting the capabilities and performance of the proposed hybrid stacked GRU-LSTM algorithm.

**Figure 9:**
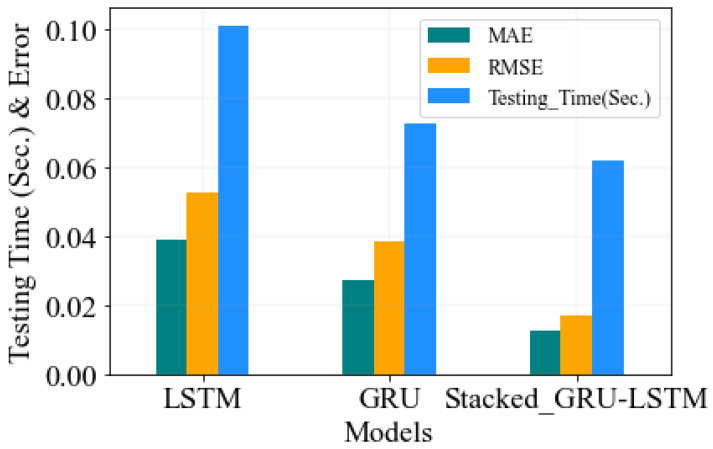
Performance of predictive analysis Bragg wavelength in terms of MAE, RMSE, and testing time using stacked GRU-LSTM with other models.

Figure 9 illustrates the results of the predictive performance analysis of three deep learning models, stacked GRU-LSTM, GRU, and LSTM, in the detection of peak wavelength values of overlapped FBG sensors. These models were evaluated using performance metrics such as MAE, RMSE, and testing time. The evaluation metrics were computed and tested using the same amount of spectral training datasets and under identical environmental conditions. The validity of the well-trained deep learning models was assessed using new, unseen test sets.

Based on the predictive analysis of peak wavelength values of overlapped FBG sensors, the MAE values varied among the three models. The stacked GRU-LSTM model achieved the lowest MAE value of 0.01248531, indicating a higher level of accuracy in its predictions. Whereas, the LSTM model displayed the highest MAE value of 0.0391585, implying a relatively lower accuracy in its predictions. In terms of the RMSE values, the stacked GRU-LSTM model achieved the lowest value of 0.017185, indicating a higher precision in its predictions. Conversely, the LSTM model yielded the highest RMSE value of 0.052951, suggesting a comparatively lower precision. Furthermore, the testing time for making predictions varied among the models. The stacked GRU-LSTM model displayed the lowest testing time value of 0.062, indicating a shorter processing duration for making predictions. In contrast, the LSTM model exhibited the highest testing time value of 0.101, implying a relatively longer processing duration. Overall, the results presented in Figure 9 highlight the superior performance of the stacked GRU-LSTM model compared to the GRU and LSTM models in terms of MAE, RMSE, and testing time. This model consistently achieved better accuracy, precision, and faster processing in predicting the peak wavelength values of overlapped FBG sensors.

**Figure 10:**
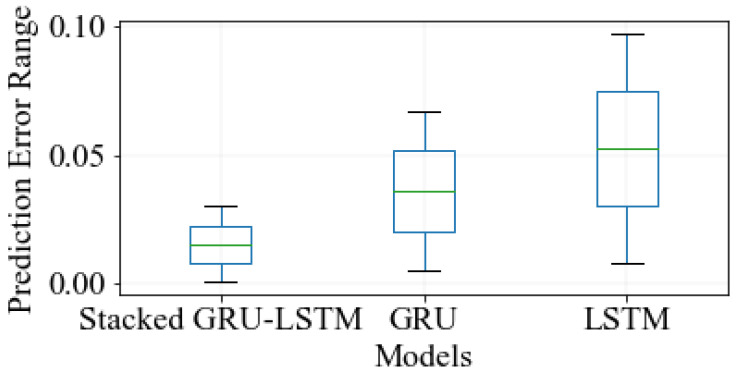
Performance comparison of measured error distribution between the actual and the measured wavelengths in between stacked GRU-LSTM, GRU, and LSTM models.

To evaluate the performance of the proposed hybrid stacked GRU-LSTM model, a comprehensive comparison was conducted with various models based on the error distribution between the actual and predicted strain-sensing signals. This evaluation utilized a new experimental unseen test set, ensuring unbiased and robust analysis. The measured error distribution was carefully examined and compared, offering insights into the accuracy and effectiveness of the proposed model compared to others. The comparison results are visually illustrated in Figure 10, providing a clear reference for evaluating the model’s suitability for strain-sensing applications. This particular evaluation, combined with the visual representation, enhances the credibility and reliability of the comparison, strengthening our understanding of the proposed hybrid stacked GRU-LSTM model’s performance. The measurement error distribution was calculated by determining the difference between the actual central wavelength value and the predicted central wavelength value. To provide a comprehensive understanding of this error distribution, Figure 10 presents a statistical representation that includes information on maximum errors, minimum errors, and the mean of errors in the prediction error distributions. Accordingly, the proposed hybrid stacked GRU-LSTM model excels across these performance indicators. It achieved a maximum error, minimum error, and mean error of 0.029, 0.0003, and 0.029, respectively. This outstanding performance outshines that of the other algorithms under consideration, underscoring the superior predictive ability of the proposed model. Its ability to accurately estimate the central wavelength value showcases its effectiveness and reliability compared to alternative models. In contrast, when comparing the maximum error values for the GRU and LSTM models, we found that they are 0.067 and 0.098, respectively. This comparison highlights that the proposed stacked GRU-LSTM model outperforms the other methods in measuring the central wavelength, as it achieved a significantly smaller maximum error. Impressively, the maximum error for the proposed model was only 0.029, demonstrating its superior accuracy and reliability in predicting strain-sensing signals. Based on the results depicted in Figure 10, it is evident that the proposed hybrid stacked GRU-LSTM model excels in terms of the error distribution analysis between the actual and predicted strain-sensing signals. This comparison proves the model’s remarkable performance, as it consistently outperformed other algorithms by achieving smaller maximum errors and delivering higher precision in measuring the central wavelength. These findings validate the superiority of this proposed model and accentuate its potential to improve the accuracy and reliability of strain-sensing applications.

In Figure 3, it is shown that the training phase of the stacked GRU-LSTM method has been completed, resulting in the acquisition of a well-trained model. This model, built upon the stacked GRU-LSTM architecture, has undergone rigorous training to acquire proficiency in capturing intricate temporal patterns within sequential data. To validate the reliability and efficacy of the trained model in handling unexplored and unseen data, a comprehensive testing process was conducted. This involved subjecting the well-trained stacked GRU-LSTM model to novel and original experimental spectral testing sets that were not part of the training datasets. By employing these new experimental test datasets, researchers can assess the model’s ability to generalize and make accurate predictions beyond the context of its training data. This testing phase serves as a critical step in measuring the model’s robustness and its potential to perform effectively in real-world scenarios.

**Figure 11:**
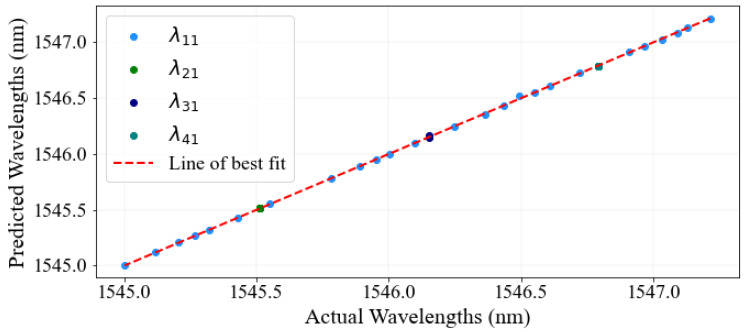
Scatter plot points of predicted vs. actual peak wavelength values for four FBG sensor signals using the stacked GRU-LSTM method.

The analysis of the model’s performance is presented in Figure 11; the estimated wavelengths of four FBG sensor spectra of the test sets are demonstrated in comparison to their actual wavelengths by utilizing the stacked GRU-LSTM technique. The figure demonstrates the relationship between the actual and predicted wavelength values, with the *x*-axis representing the actual values and the *y*-axis representing the predicted values of the test sets. The red line serves as an indication of the proximity of the ideal point to the line of best fit. Upon closer examination of the figure, it becomes evident that the actual and predicted peak wavelengths validate a strong correlation and are aligned more closely with the line of best fit in the case of the stacked GRU-LSTM model. Particularly, when evaluating the RMSE values, it is observed that the stacked GRU-LSTM model achieved the lowest RMSE value of 0.017185, indicating a minimal level of detection error.

**Figure 12:**
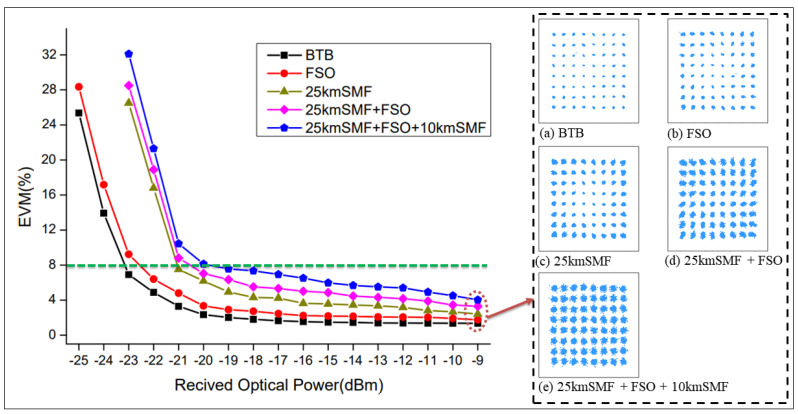
The measurement output of performance parameters for the communication system at different distances of the transmission channels.

The second case pertains to demonstrating the results analysis of the optical communication system. The performance evaluation of the integrated fiber/FSO communication system involved analyzing key parameters such as EVM and constellation diagrams. This performance analysis system involved evaluating the EVM and constellation diagram values, which consider various transmission distances and FSO links. To demonstrate the results, Figure 12 shows the EVM curve and constellation diagram for various scenarios, including back-to-back (BTB), FSO, 25 km SMF, 25 km SMF with FSO, and 25 km SMF combined with an additional 10 km SMF integrated with FSO transmission channels. Throughout all scenarios, the received optical power level was set at −9 dBm, resulting in corresponding EVM values of 1.36%, 1.76%, 2.41%, 3.3%, and 4.03%, respectively. The higher EVM values could be due to factors such as dispersion and minor reflections within the fiber links. However, the constellation diagrams obtained from the measurements demonstrate the successful propagation of signals even when integrating different transmission channels. This important observation indicates the effectiveness of the proposed hybrid fiber sensor/FSO communication network, which incorporates sensor integration. It also signifies the network’s ability to overcome geographical limitations and achieve improved performance while extending the transmission distance. Overall, the communication results provide a comprehensive evaluation of the integrated fiber sensor/FSO communication system. The utilization of EVM measurements and constellation diagrams confirms the system’s capability to maintain reliable signal transmission, even in challenging conditions. The integration of fiber and FSO technologies within the proposed hybrid network shows its potential as a viable solution, offering enhanced performance, extended transmission distance, and the ability to overcome geographical barriers.

## 6. Conclusions

In this paper, a new bidirectional integration approach was introduced to combine fiber sensor/FSO communication with an IWDM techniques-based long-distance strain-sensing FBG sensor system. By implementing CWDM, the system can transmit optical communication and FOS sensing signals simultaneously, leading to the creation of a system that is highly capable, flexible, and cost-effective. This bidirectional structure is a preferential option and an optimal solution for ensuring the reliability and stability of a long-distance FBG sensor system. Extensive research and experimentation support this conclusion. Additionally, a hybrid approach that merges stacked GRU and LSTM deep learning algorithms was proposed. This hybrid stacked GRU-LSTM model outperforms individual GRU and LSTM models in peak wavelength detection accuracy. This hybrid approach greatly enhances the precision of peak wavelength detection in FBGs that have overlapping spectra.

## Data Availability

Not applicable.
